# MiR-1-3p Inhibits Lung Adenocarcinoma Cell Tumorigenesis via Targeting Protein Regulator of Cytokinesis 1

**DOI:** 10.3389/fonc.2019.00120

**Published:** 2019-03-01

**Authors:** Tao Li, Xiuxiu Wang, Lijun Jing, Yu Li

**Affiliations:** Department of Respiratory Diseases, Qilu Hospital of Shandong University, Jinan, China

**Keywords:** lung adenocarcinoma, miR-1-3p, protein regulator of cytokinesis 1, malignant behavior, mechanism

## Abstract

Lung adenocarcinoma (LUAD) is one of the most lethal malignancies, posing a threat to human health. However, the molecular mechanisms underlying LUAD development remain largely unknown. In this study, we found that miR-1-3p was significantly downregulated in human LUAD tissues and cell lines and played an inhibitory role in LUAD cell tumorigenesis, as evidenced by the significantly reduced viability, migration, and invasion of LUAD cells in response to miR-1-3p overexpression. Mechanistically, microRNA (miR)-1-3p physically interacted with the 3′-untranslated region (UTR) of protein regulator of cytokinesis 1 (PRC1) mRNA, leading to downregulation of PRC1. Overexpression of PRC1 reversed the inhibitory effects of miR-1-3p on LUAD cell tumorigenesis, suggesting that the miR-1-3p/PRC1 axis is majorly involved in suppressing LUAD development and progression. Consistently, PRC1 was dramatically induced in LUAD tissues and cell lines as well as associated with a poor prognosis in LUAD patients. Taken together, our study identified the miR-1-3p/PRC1 axis as an important regulatory mechanism contributing to LUAD inhibition and provided valuable clues for the future development of therapeutic strategies against LUAD.

## Introduction

Lung adenocarcinoma (LUAD) is the most common subtype of non-small cell lung cancer (NSCLC), accounting for 80–85% of all lung cancers; worldwide, approximately 40% of all lung cancer patients are diagnosed with LUAD (1). Compared to other subtypes of NSCLC, LUAD has a higher incidence and a shorter survival time among patients, with a 5-year survival rate as low as 10–15% (2, 3); thus, LUAD poses a serious threat to human health. Currently, chemotherapy is a relatively effective therapeutic option for NSCLC (4). However, the existence and development of intrinsic or acquired chemoresistance greatly limit the application of chemotherapy in cancer treatment. Therefore, there is still an urgent need to develop novel therapeutic strategies against LUAD that are based on the mechanisms underlying the development and progression of LUAD.

MicroRNAs (miRNAs) are a class of small endogenous non-coding RNA molecules (~22 nucleotides) found in animals and plants that are responsible for the degradation or translation repression of mRNAs by binding to the 3′-untranslated region (UTR) of target mRNAs (5). A variety of miRNAs have been identified as novel biomarkers or promising therapeutic targets of human malignant tumors. Among them, miR-1-3p plays an antitumor role in multiple cancer types, including rhabdomyosarcoma as well as lung, thyroid, prostatic, bladder, colorectal, and hepatocellular carcinomas (6–11). However, the role of miR-1-3p in LUAD has not yet been investigated. In addition, the association between miR-1-3p and its target genes may deepen our understanding of the molecular mechanisms contributing to LUAD development, thus facilitating the discovery of improved therapies for LUAD.

The microtubule-associated protein regulator of cytokinesis 1 (PRC1) has been found to be majorly involved in the organization of antiparallel microtubules in the central spindle during cytokinesis. The human PRC1 gene, located on chromosome 15q26.1, encodes a 620-amino-acid protein with a molecular weight of 71 kDa (3). An abnormally high expression of PRC1 has been observed in breast cancer (12), bladder cancer (13), hepatocellular carcinoma (14), and pancreatic cancer (15), which suggests a promotive role of PRC1 in tumorigenesis. However, it remains largely unknown whether there is a functional association between miR-1-3p and the regulation of PRC1 in LUAD. In this study, we examined the role of miR-1-3p in LUAD growth and metastasis as well as the underlying mechanism.

## Materials and Methods

### Tissue Samples From LUAD Patients

Human LUAD tissues were collected from LUAD patients undergoing pulmonary resection or bronchoscopy biopsy, and normal tissues adjacent to cancer were collected from LUAD patients undergoing pulmonary resection at Qilu Hospital between May and September 2018. None of the patients had received chemotherapy or radiotherapy prior to surgery. All the fresh samples were stored in RNAlater Stabilization Solution (Ambion) at −80°C until use. This study was approved by the Ethics Committee of Shandong University, and written informed consent was obtained from all patients prior to enrollment in the present study. The clinicopathological characteristics of the patients are shown in Supplementary Table 1.

### Cell Culture

Three LUAD cell lines (A549, H1299, and H1975 cells) and a human alveolar epithelial cell line (HPAEpiC) were purchased from the Cell Bank of the Type Culture Collection of the Chinese Academy of Sciences (Shanghai, China). The cells were maintained in RPMI 1640 medium (Gibco, USA) containing 10% fetal bovine serum (Gibco, USA), 100 U/mL penicillin, and 100 μg/mL streptomycin in a humidified atmosphere of 5% CO_2_ at 37°C. Cells in the exponential phase of growth were used for the following experiments.

### Construction of the miR-1-3p Overexpression Cell Lines

The pre-miR-1-3p sequences were synthesized by Biosune Biotechnology Company (Shanghai, China) and cloned into the lentiviral vector pGIPZ. Lentivirus was produced in HEK293T cells using the packaging vectors psPAX2 and pMD2.G. The cells were infected with lentivirus for 24 h and then cultured for 1 week in medium containing 2 μg/mL puromycin (Merck Millipore, USA) for screening to acquire cells with stable expression of miR-1-3p.

### Transient Transfection

The miR-1-3p mimic and its negative control (NC) were chemically synthesized by GenePharma Co., Ltd. (Shanghai, China). The cells were transiently transfected with 50 nM miR-1-3p mimic or 50 nM NC (Boshang, Inc., China) using Lipofectamine 2000 (Invitrogen; Thermo Fisher Scientific, Inc.), according to the manufacturer's protocol. The cells were harvested at 24 or 48 h after the transfection. The NC was a scrambled oligonucleotide that does not encode any known miRNA. The transfection efficiency was confirmed by detecting the miR-1-3p expression level using the SYBR green (Takara)-based real-time quantitative polymerase chain reaction (qPCR) system.

### RNA Isolation and qPCR

Total RNA was extracted from the cells using Trizol reagents (Invitrogen; Thermo Fisher Scientific, Inc.), according to the manufacturer's instructions. The cDNA of miRNA was synthesized with the One Step PrimeScript miRNA cDNA Synthesis Kit (Takara Biotechnology, Co., Ltd., Dalian, China). qPCR was performed using the SYBR green Premix Ex Taq II (Takara Biotechnology, Co., Ltd.) with the Step One Plus Real-Time PCR System (Applied Biosystems; Thermo Fisher Scientific, Inc.). The expression of U6 was used as an internal control. The primers for miR-1-3p are indicated in Supplementary Table 2.

### Western Blot Analysis

The cells were lysed in ice-cold RIPA lysis buffer, and the cell lysates were obtained by centrifugation at 12,000 rpm and 4°C for 10 min. The protein concentration was determined using the bicinchoninic acid method. The protein samples (5–10 μg) were separated by sodium dodecyl sulfate–polyacrylamide gel electrophoresis, followed by transfer to polyvinylidene difluoride membranes, and then immunoblotted with the indicated antibodies. After blocking with 5% nonfat milk, the membranes were incubated with the respective primary antibody overnight at 4°C, followed by incubation with the horseradish peroxidase-coupled secondary antibody for 1 h at room temperature. The protein bands were visualized using enhanced chemiluminescence reagents (PerkinElmer) with an ImageQuant LAS 4000 system (GE Healthcare Life Sciences). The following antibodies were used: anti-PRC1, anti-fibronectin, anti-N-cadherin, anti-vimentin, and anti-β-actin (Cell Signaling Technology).

### 3-(4,5-Dimethylthiazol-2-yl)-2,5-Diphenyltetrazolium Bromide (MTT) Assay

The cells were seeded into 96-well plates at a density of 2,000 cells/well and grown for 5 days. After the addition of 100 μL of 5 mg/mL MTT solution, the cells were incubated for an additional 4 h at 37°C, and then the supernatant was removed and dissolved in 100 μL of dimethyl sulfoxide (Sigma-Aldrich). Cell viability was assessed on the 1st, 2nd, 3rd, 4th, and 5th day. The absorbance of each well was measured in triplicate using an iMark Microplate Absorbance Reader (Bio-Rad).

### Luciferase Reporter Assay

Luciferase reporter constructs containing wild-type (WT) or mutant PRC1 3′-UTR (pmirGLO-PRC1-WT or pmirGLO-PRC1-mut, respectively) were generated by GenePharma Inc. (Shanghai, China). The cells were cotransfected with 25 ng of PRC1 3′-UTR reporter constructs and 20 nM miR-1-3p mimic using Lipofectamine 2,000 (Invitrogen) in 24-well plates. At 24 h after transfection, luciferase assays were performed using the Dual-Luciferase reporter assay system (Promega). *Renilla* luciferase activity was used to normalize the luciferase activity of the PRC1 3′-UTR reporter constructs.

### *In vivo* Tumorigenicity Assays

Four-week-old male BALB/c nude mice were purchased from the Shanghai Laboratory Animal Center of the Chinese Academy of Sciences (Shanghai, China). The mice were randomly divided into two groups and injected subcutaneously with A549 cells (2 × 10^6^ cells/mouse, *n* = 5 mice/group) that were infected with either control lentivirus or miR-1-3p-overexpressing lentivirus. Tumor growth was monitored by measuring the tumor diameter. Tumor volume was calculated according to the formula TV (cm^3^) = a × b^2^ × π/6, where a is the longest diameter and b is the shortest diameter. The mice were sacrificed after 3 weeks, and then the tumors were excised and weighed. All animal experiments were approved by the Shandong University Animal Care and Use Committee.

### Bioinformatics Analyses

PRC1 genetic alterations and copy number variation in LUAD were retrieved from the cBioPortal for Cancer Genomics (http://www.cbioportal.org/) (16, 17). The Cancer Genome Atlas RNA expression data of LUAD tissues were processed and analyzed by the Cancer Genomics Browser (https://xena.ucsc.edu/welcome-to-ucsc-xena/) (18). The PRC1 expression levels and copy number variation were analyzed by Proteinatlas (https://www.proteinatlas.org/), Oncomine (www.oncomine.org) (19), and Gene Expression Profiling Interactive Analysis (http://gepia.cancer-pku.cn/) in LUAD and normal lung tissues via immunohistochemistry. Kaplan–Meier plots (http://kmplot.com/analysis/) (20) were used to analyze the overall survival of the LUAD patients.

### Statistical Analysis

Statistical analysis was performed using GraphPad Prism 6.0 (GraphPad Software, La Jolla, CA, USA). Data are expressed as the mean ± standard deviation. Comparison between two groups was performed using the Student's *t*-test or the Mann-Whitney *U*-test. The correlation between the expression levels of miR-1 and PRC1 was analyzed using Pearson's correlation analysis. LUAD tissues with lower miR-1 and PRC1 expression than the median expression were assigned to the low-expression group, whereas those with higher miR-1 and PRC1 expression than the median expression were assigned to the high-expression group. Associations between the clinicopathological features and the expression levels of miR-1 and PRC1 were analyzed using the χ^2^ test. Overall survival curves were determined according to the Kaplan–Meier method. A *p* < 0.05 was considered statistically significant.

## Results

### MiR-1-3p Is Downregulated in Human LUAD Tissues and Cell Lines

**Figure 1:**
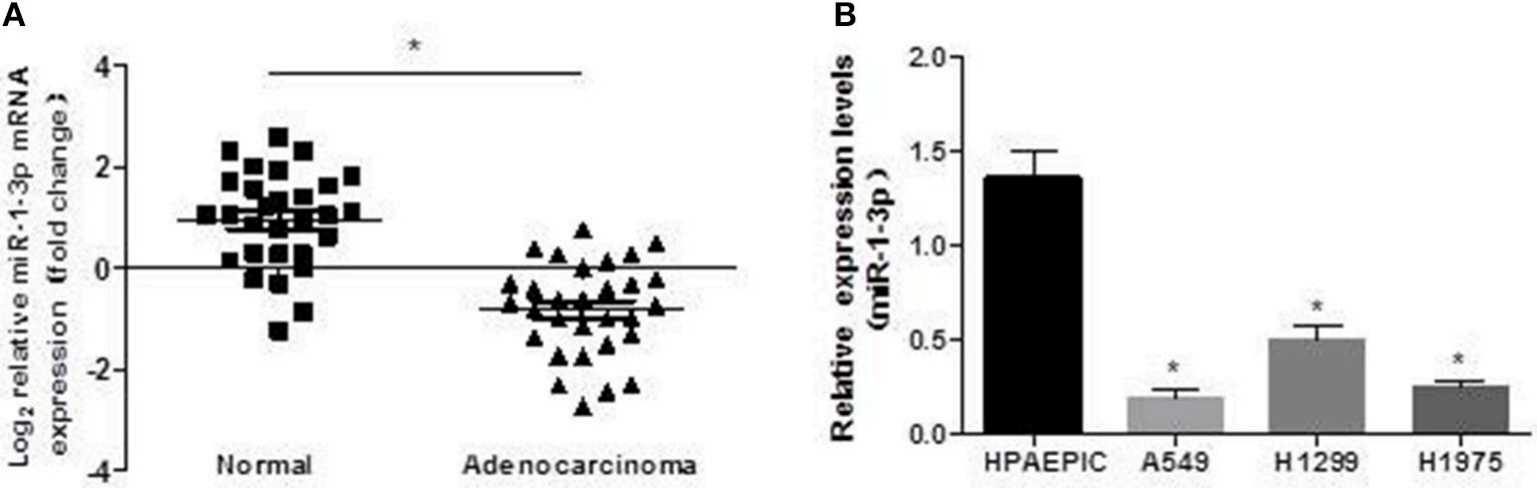
Expression pattern of miR-1-3p in human LUAD tissues and cell lines. qPCR was performed to determine the expression levels of miR-1-3p in human LUAD tissues **(A)** and cell lines **(B)**, as indicated. ^*^P < 0.05 in **(A)** (*n* = 30); ^*^P < 0.05 vs. HPAEpiCs in **(B)** (*n* = 3).

To investigate the possible role of miR-1-3p in LUAD development, we first examined the expression levels of miR-1-3p in human LUAD tissues and cell lines. As shown in Figure 1A, miR-1-3p expression was significantly decreased in LUAD tissues, compared with the matched adjacent normal lung tissues. Similarly, marked downregulation of miR-1-3p was also observed in the human LUAD cell lines A549, H1299, and H1975, compared with the normal HPAEpiCs (Figure 1B). These *in vivo* and *in vitro* results suggest that miR-1-3p may play an inhibitory role in LUAD development.

### Overexpression of miR-1-3p Suppresses LUAD Cell Viability *in vitro*

**Figure 2:**
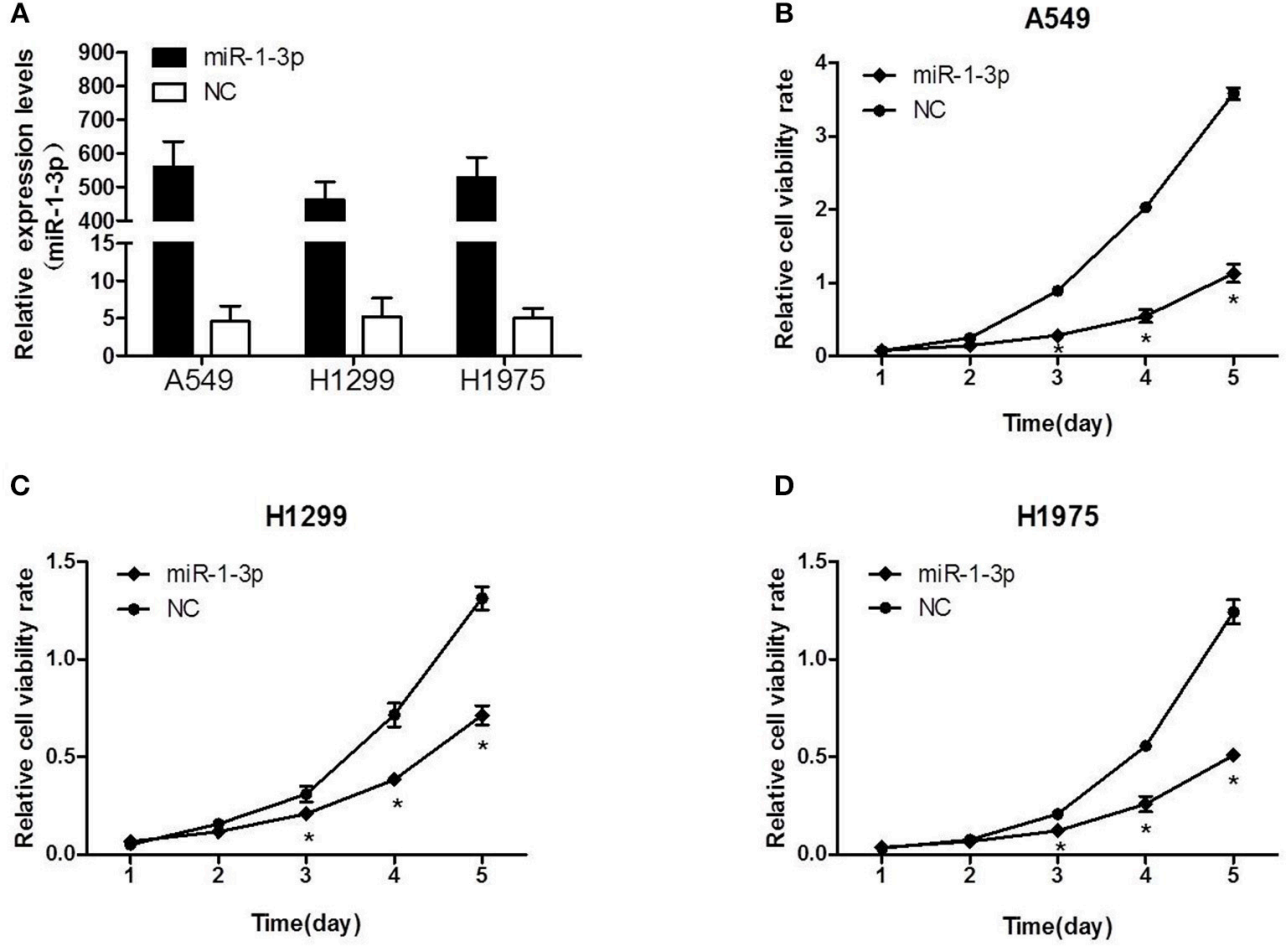
The effect of miR-1-3p overexpression on LUAD cell viability. **(A)** qPCR was performed to validate the overexpression efficiency of miR-1-3p in A549, H1299, and H1975 cells. **(B–D)** The MTT assay was performed to measure the viability of miR-1-3p-overexpressing A549, H1299, and H1975 cells. ^*^P < 0.05 vs. the corresponding negative control (NC) groups (*n* = 3).

Next, we sought to investigate whether miR-1-3p indeed plays a role in suppressing LUAD development using a gain-of-function assay. As shown in Figure 2A, transfection of the miR-1-3p mimic led to a dramatic increase in miR-1-3p expression in the LUAD cell lines A549, H1299, and H1975. Importantly, overexpression of miR-1-3p significantly inhibited the viability of these three cell lines in a time-dependent manner, compared with the NC groups (Figures 2B–D). These results demonstrate that miR-1-3p is sufficient to suppress LUAD cell growth *in vitro*.

### Overexpression of miR-1-3p Inhibits LUAD Cell Migration and Invasion *in vitro*

**Figure 3:**
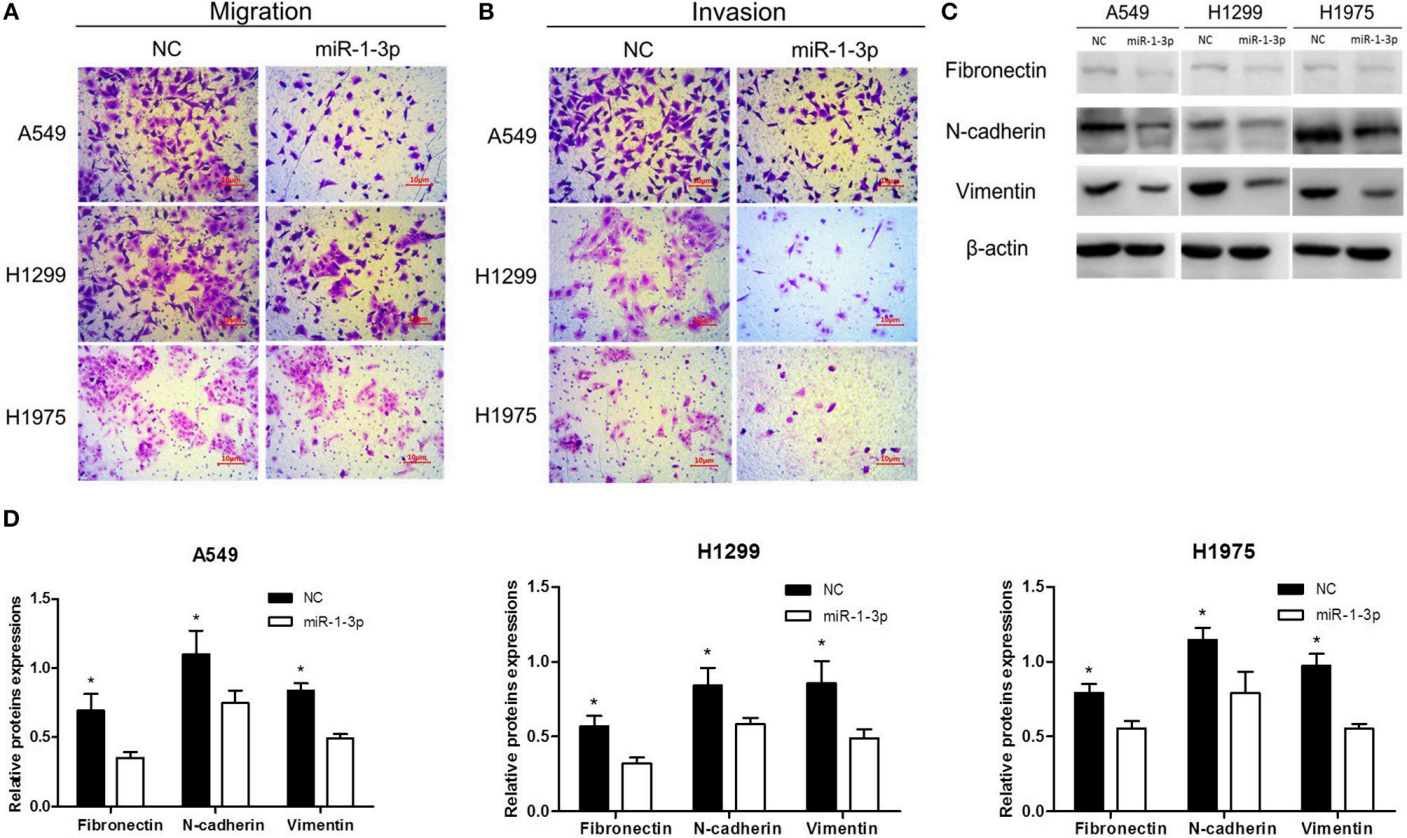
The effect of miR-1-3p overexpression on LUAD cell migration and invasion as well as epithelial-mesenchymal transition. Transwell assays for migration **(A)** and invasion **(B)** of miR-1-3p-overexpressing LUAD cells. **(C)** Western blot assay for the expression of the indicated mesenchymal markers. β-Actin was used as an internal control. **(D)** Quantification of the western blot assay results shown in **(C)**. ^*^P < 0.05 vs. miR-1-3p groups in **(C)** (*n* = 3).

To further investigate whether miR-1-3p inhibits LUAD progression, Transwell assays were performed to examine the effects of miR-1-3p overexpression on LUAD cell migration and invasion. As shown in Figures 3A,B, overexpression of miR-1-3p resulted in a significant decrease in LUAD cell migration and invasion abilities, compared with the NC groups. Consistently, overexpression of miR-1-3p markedly suppressed epithelial-mesenchymal transition (EMT), a process contributing to tumor metastasis, as evidenced by downregulation of the mesenchymal markers fibronectin, N-cadherin, and vimentin (Figures 3C,D). These data suggest that miR-1-3p overexpression may suppress LUAD progression through reducing LUAD cell migration and invasion as well as inhibiting EMT in these cells.

### miR-1-3p Inhibits Xenograft Tumor Growth of LUAD Cells

**Figure 4:**
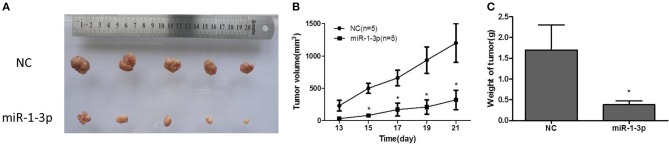
The effect of miR-1-3p overexpression on tumorigenesis *in vivo*. **(A)** Overexpression of miR-1-3p reduced LUAD cell-derived tumor growth in a xenograft model. **(B)** Summary of the tumor growth data; the error bars indicate the standard deviation. **(C)** miR-1-3p overexpression results in a decline of tumor weight. ^*^P < 0.05.

To further explore whether miR-1-3p overexpression could suppress LUAD growth *in vivo*, human LUAD A549 cells with and without miR-1-3p overexpression were subcutaneously inoculated into nude mice. At 1 week after inoculation, all mice had developed detectable tumors. However, at 3 weeks after inoculation, the mice bearing tumors with miR-1-3p overexpression demonstrated a dramatic decrease in the tumor size and weight (Figures 4A–C), compared to the control groups. These results show that overexpression of miR-1-3p inhibits tumorigenesis *in vivo*.

### PRC1 Is a Direct Target Gene of miR-1-3p

**Figure 5:**
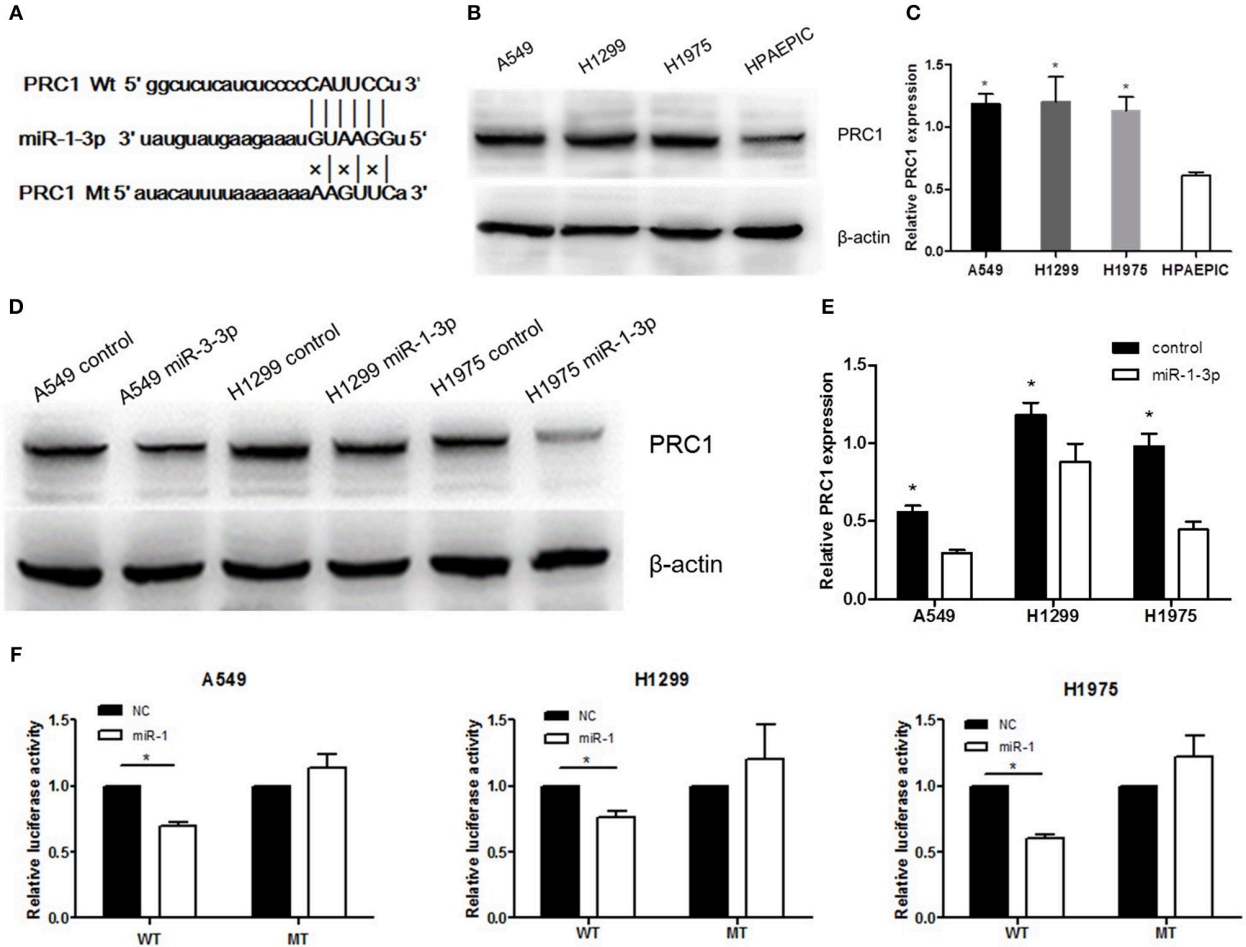
PRC1 is a direct target of miR-1-3p in LUAD cells. **(A)** The putative miR-1-3p-binding sites in PRC1 3′-UTR and the mutated binding sites are shown. **(B,D)** Western blot analysis of PRC1 expression in the nontransfected negative control (NC)- or miR-1-3p-transfected LUAD cells. β-Actin was used as an internal control. **(C)** Quantification of the western blot assay results shown in **(B)**. ^*^P < 0.05 vs. HPAEpiCs (*n* = 3). **(E)** Quantification of the western blot assay results shown in **(D)**. ^*^P < 0.05 vs. the miR-1-3p groups (*n* = 3). **(F)** Luciferase reporter assay for LUAD cells transfected with wild-type (WT) or mutated (MT) pGL3-3′-UTR. The luciferase activity was normalized to *Renilla* luciferase activity. ^*^P < 0.05 (*n* = 3).

To further examine the mechanism underlying miR-1-3p-mediated suppression of LUAD development and progression, we employed the TargetScan computational algorithm to predict the target genes of miR-1-3p (21). The results indicated complementary base-pairing between miR-1-3p and the 3′-UTR of PRC1 (Figure 5A), suggesting that PRC1 may be a target gene of miR-1-3p. To verify this finding, we detected the expression of PRC1 in LUAD cells. As shown in Figures 5B,C, the protein expression of PRC1 was markedly induced in LUAD cells, compared with normal HPAEpiCs, consistent with the expression pattern of miR-1-3p in LUAD tissues and cells. Importantly, miR-1-3p overexpression led to downregulation of PRC1 in LUAD cells (Figures 5D,E), confirming that PRC1 is a downstream target of miR-1-3. To determine whether PRC1 is directly targeted by miR-1-3p, we performed a mutation assay through introducing a PRC1 3′-UTR mutation in the pmirGLO vector. The results demonstrated that the PRC1 3′-UTR mutation had no significant effect on the luciferase activity in miR-1-3p-transfected LUAD cells, compared with that in the NC-transfected cells (Figure 5F), suggesting that WT PRC1 3′-UTR is essential for the function of miR-1-3p. Taken together, our data show that PRC1 is a direct downstream target gene of miR-1-3p.

### PRC1 Is Induced in LUAD Tissues and Cell Lines

**Figure 6:**
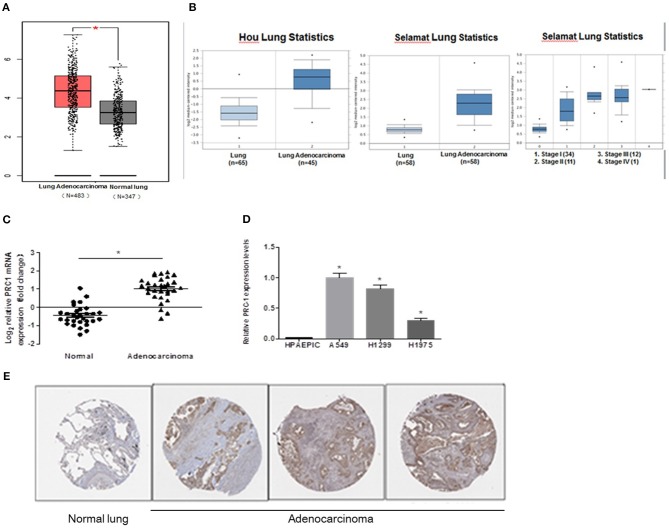
Expression pattern of PRC1 in LUAD tissues and cell lines. **(A)** The data of copy number variation in LUAD from The Cancer Genome Atlas cohort. **(B)** The mRNA expression of PRC1 in different TNM staging groups in Oncomine. **(C)** qPCR analysis of PRC1 expression in LUAD tissues (*n* = 30). **(D)** qPCR analysis of PRC1 expression in LUAD cells and HPAEpiCs. **(E)** Representative images of the immunohistochemical staining of PRC1 from The Human Protein Atlas in LUAD and normal lung tissues. ^*^P < 0.05 in **(C)** (*n* = 30); ^*^P < 0.05 vs. HPAEpiCs in **(D)** (*n* = 3).

To determine whether PRC1 contributes to LUAD development and progression, we examined the expression profile of PRC1 in LUAD tissues using the publicly accessible database Oncomine. As shown in Figure 6A, the mRNA expression levels of PRC1 were significantly enhanced in the LUAD tissues, compared with the normal lung tissues. In addition, we also analyzed the mRNA expression of PRC1 in LUAD tissues using two microarray datasets from the Hou and Selamat lung cancer groups, which were downloaded from Oncomine. The results demonstrated that the mRNA expression of PRC1 was significantly induced in the LUAD tissues of these groups and positively correlated with the tumor, lymph node, metastasis (TNM) staging of LUAD (Figure 6B). These findings were further confirmed by our mRNA expression data of PRC1 and the immunohistochemical staining of PRC1 in human LUAD tissues and matched adjacent normal lung tissues (Figures 6C,E). For the *in vitro* study, the mRNA levels of PRC1 were dramatically increased in the LUAD cell lines A549, H1299, and H1975, compared to those in normal HPAEpiCs (Figure 6D). Collectively, these results suggest that PRC1 may be involved in LUAD development and progression.

### Correlation of miR-1-3p/PRC1 and Clinicopathological Characteristics of LUAD Patients

**Figure 7:**
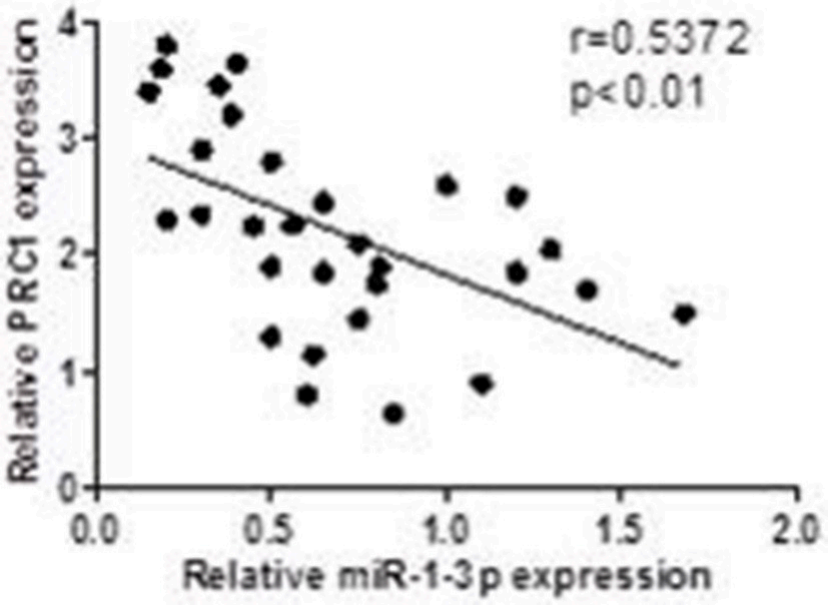
Association between the miR-1-3p and PRC-1 levels in LUAD patients. The PRC1 and miR-1 levels were determined by qPCR in LUAD tissues and matched adjacent normal tissues (*n* = 30).

To investigate whether the miR-1-3p/PRC1 axis plays a role in LUAD development, we first analyzed the association between the miR-1-3p and PRC1 levels in LUAD tissues. The results revealed that the miR-1-3p levels were negatively correlated with the PRC1 mRNA expression (*r* = −0.5858; *P* < 0.01; Figure 7) in the LUAD tissues. Importantly, low levels of miR-1-3p and high levels of PRC1 were strongly associated with the TNM stage, lymph node metastasis, and distant metastasis (Table 1). These data suggest that LUAD development may be at least partially attributable to the miR-1-3p/PRC1 axis.

**Table 1:** The clinicopathological characteristics of 30 lung adenocarcinoma patients.

**Clinicopathological features**	***n***	**Percent (%)**	**PRC1 expression**		***p***	**miR-1-3p expression**		***p***
			**Low (*n* = 9)**	**High (*n* = 21)**		**Low (*n* = 18)**	**High (*n* = 12)**	
**GENDER**								
Male	19	63.33	7	12	0.419	11	9	0.694
Female	11	36.67	2	9	–	7	3	–
**AGE (YEARS)**								
≤60	13	43.33	6	7	0.123	8	5	1.000
>60	17	56.67	3	14	–	10	7	–
**TUMOR SIZE**								
T1 and T2	6	20.00	1	5	0.637	4	2	1.000
T3 and T4	24	80.00	8	16	–	14	10	–
**TNM STAGE**								
I and II	7	23.33	5	2	0.014	1	6	0.009
III and IV	23	76.67	4	19	–	17	6	–
**LYMPHATIC METASTASIS**								
Negative	12	40.00	8	4	0.001	2	10	0.000
Positive	18	60.00	1	17	–	16	2	–
**DISTANT METASTASIS**								
M0	12	40.00	7	5	0.013	3	9	0.002
M1	18	60.00	2	16	–	15	3	–

*TNM, tumor, lymph node, metastasis stage*.

### miR-1-3p Inhibits LUAD Cell Metastasis via PRC1

**Figure 8:**
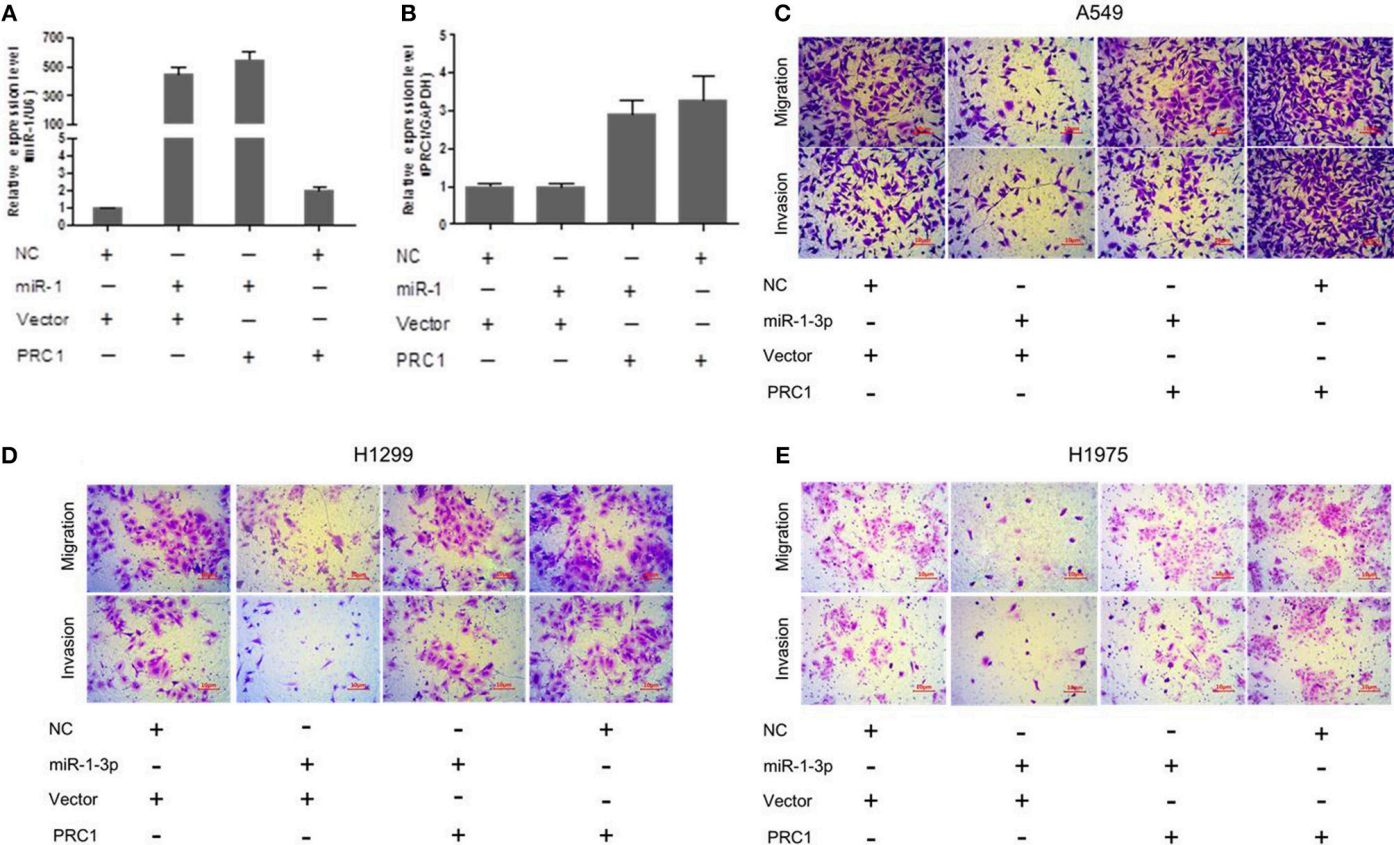
miR-1-3p inhibits LUAD cell migration and invasion via PRC1. **(A,B)** qPCR analysis of the transfection efficiency of the miR-1-3p mimic and the PRC1-overexpression plasmid in LUAD cells. **(C–E)** Transwell assays for migration and invasion of LUAD cells transfected with the miR-1-3p mimic and/or the PRC1-overexpression plasmid.

To determine whether miR-1-3p-mediated suppression of PRC1 expression is a major mechanism inhibiting LUAD development and progression, we cotransfected LUAD cells with miR-1-3p and a PRC1-overexpression plasmid for Transwell assays. The plasmid transfection efficacy of miR-1-3p and PRC1 was validated in Figures 8A,B. We found that miR-1-3p significantly inhibited the migration and invasion of three LUAD cell lines and that the inhibitory effects of miR-1-3p were markedly reversed by PRC1 overexpression (Figures 8C–E), suggesting that miR-1-3p inhibits LUAD cell metastasis in a PRC1-depedent manner and the miR-1-3p/PRC1 axis is majorly involved in LUAD development and progression.

### Overexpression of PRC1 Is Associated With a Poor Prognosis in LUAD Patients

**Figure 9:**
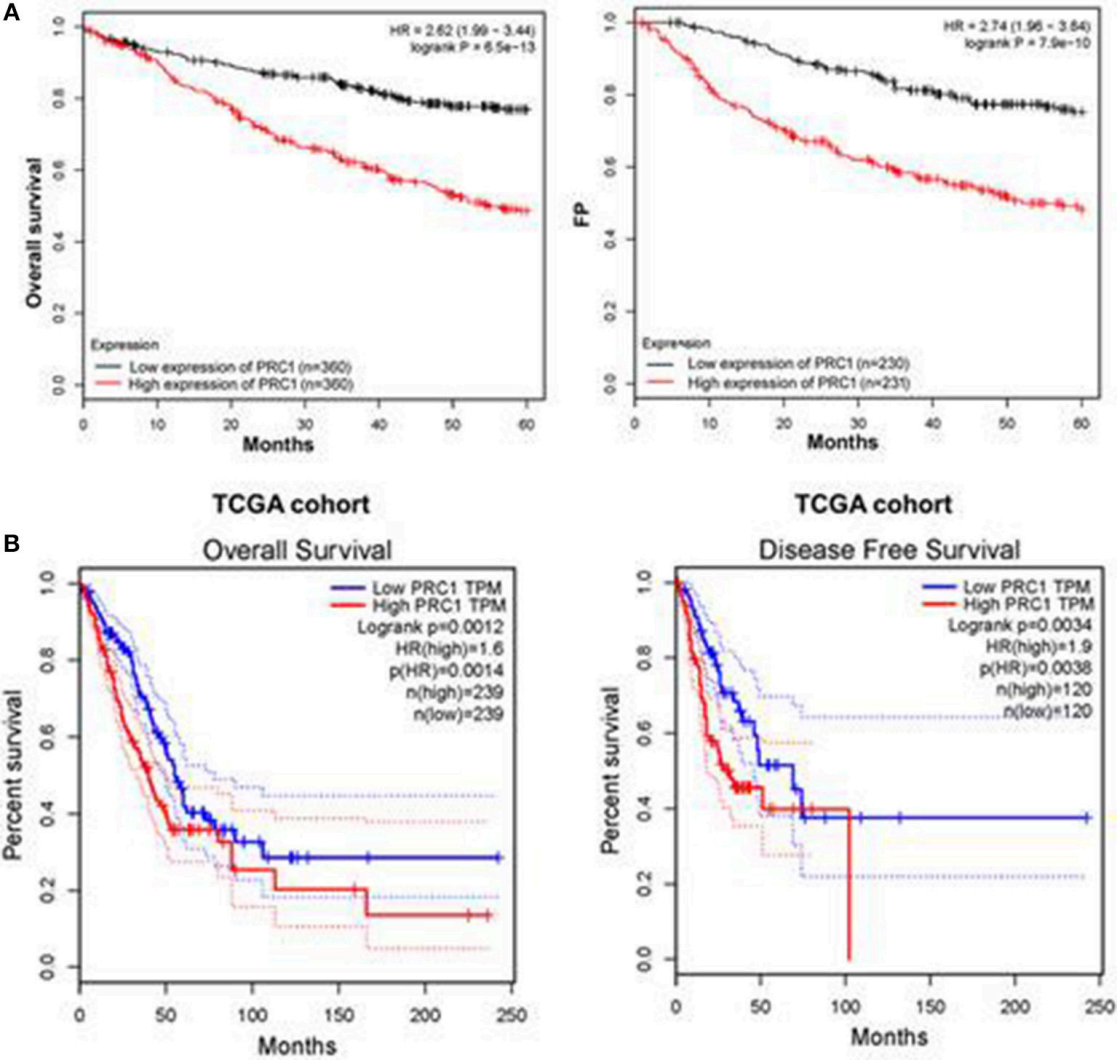
Overexpression of PRC1 is associated with a poor prognosis in LUAD patients. **(A)** The prognostic effect of PRC1 in LUAD patients was evaluated using Kaplan–Meier plots. **(B)** The overall and disease-free survival rates in LUAD patients with different PRC1 expression patterns were evaluated for The Cancer Genome Atlas (TCGA) cohort.

MiR-1-3p functions through suppressing PRC1 expression, suggesting a promotive role of PRC1 in LUAD development. To confirm this, we examined the prognostic effect of PRC1 in LUAD patients from a public database by performing Kaplan–Meier analysis (http://www.kmplot.com). The results showed that the LUAD patients with a higher mRNA expression of PRC1 had shorter overall and disease-free survival times than those with a lower mRNA expression of PRC1 (Figures 9A,B). These data suggest that PRC1 overexpression may serve as a biomarker of a poor prognosis for LUAD patients, further supporting our findings that miR-1-3p plays a key role in inhibiting LUAD development through targeting PRC1.

## Discussion

Carcinogenesis of LUAD is a complex and multistage process involving the regulation of a wide range of genes by miRNAs (22–24). Among them, miR-1-3p, a muscle-specific miRNA, has been shown to play a key role in skeletal muscle differentiation and have inhibitory effects on the growth, migration, and invasion of LUAD (25). The present study revealed that miR-1-3p was significantly downregulated in LUAD tissues and cells. Lower levels of miR-1-3p were strongly associated with a higher TNM stage, earlier lymph node metastasis, and more distant metastasis. Therefore, miR-1-3p is suggested as a tumor suppressor in LUAD. The detection of miR-1-3p expression may be a valuable tool to evaluate the invasion and metastasis of LUAD.

There are hundreds of possible target genes of miR-1-3p, among which PRC1 is a critical protein in cytokinesis and characterized as a mitotic spindle-associated cyclin-dependent kinase substrate (26). Previous studies have provided evidence that PRC1 is involved in different types of cancer (27, 28). Loss of PRC1 leads to the accumulation of bi- and multi-nucleated cells in lung cancer, which further supports its role as the major central spindle organizer in cytokinesis (29). In view of our findings that miR-1-3p overexpression inhibits LUAD cell viability, there is a possibility that the function of PRC1 in apoptosis and senescence is due to induction of miR-1-3p. In this study, we demonstrated that the function of miR-1-3p could be suppressed by dysregulated expression of PRC1. In accordance with the above-mentioned studies, we confirmed that the overexpression of PRC1 significantly promoted the viability, invasion, and migration of LUAD cells. A higher PRC1 expression was also related to a worse outcome in patients with LUAD. Because Wnt/β-catenin signaling is dysregulated in lung cancer (30) and the overexpression of Wnt proteins (Wnt1 and Wnt5a) is significantly associated with adverse outcomes in lung cancer patients (31), we speculate that the miR-1-3p/PRC1 axis participates in dysregulation of Wnt/β-catenin signaling in LUAD development (32); however, this hypothesis requires further investigation. Although our study demonstrated that the miR-1-3p/PRC1 axis is a major mechanism underlying LUAD development, we do not exclude the possibility that other miRNAs or protein regulators besides miR-1-3p/PRC1 are also involved in LUAD pathogenesis. Therefore, more research is needed.

In summary, we identified miR-1-3p as a novel regulator of PRC1 in LUAD. A high PRC1 expression correlates with a poor prognosis in LUAD patients. Thus, targeting miR-1-3p/PRC1 may be a potential therapeutic intervention for the treatment of LUAD.

## Ethics Statement

The Ethics Committee of Qilu Hospital at Shandong University approved this study [KYLL-2018 (KS)-156]. All participants in this study provided informed consent.

## Author Contributions

TL designed the questionnaire and drafted the manuscript. XW did the statistical analysis. TL and LJ did the relationship analysis and collected the data. YL conceived the study, supervised and reviewed the entire study, and edited the manuscript.

### Conflict of Interest Statement

The authors declare that the research was conducted in the absence of any commercial or financial relationships that could be construed as a potential conflict of interest.
