# Supplementary material for: MiR-1-3p Inhibits Lung Adenocarcinoma Cell Tumorigenesis via Targeting Protein Regulator of Cytokinesis 1
Source: Front Oncol. 2019 Mar 1;9:120. doi: 10.3389/fonc.2019.00120 (PMC6405482; doi:10.3389/fonc.2019.00120)
Supplement: Supplementary file 1 [file Data_Sheet_1.docx]

**Supplementary table 1：**Sequences of miR-1-3p and corresponding controls

| miRNAs | miR-1-3p Mimics | S: 5′- UGGAAUGUAAAGAAGUAUGUAU -3′；  A:5′- ACAUAC UUC UUUACAUUCCAUU -3′； |
| --- | --- | --- |
|  | Mimics negative control | S:5′- UUCUCCGAACGUGUCACGUTT -3′；  A:5′- ACGUGACACGUUCGGAGAATT -3′； |
