# Supplementary material for: MiR-1-3p Inhibits Lung Adenocarcinoma Cell Tumorigenesis via Targeting Protein Regulator of Cytokinesis 1
Source: Front Oncol. 2019 Mar 1;9:120. doi: 10.3389/fonc.2019.00120 (PMC6405482; doi:10.3389/fonc.2019.00120)
Supplement: Supplementary file 2 [file Data_Sheet_2.docx]

**Supplementary table 2：**The RT and PCR primers of miR-1-3p used in qRT–PCR analysis.

|  | RT primer | CTCAACTGGTGTCGTGGAGTCGGCAATTCAGTTGAGTACATACT |
| --- | --- | --- |
| miR-1-3p | PCR primer | F: ACACTCCAGGTGGGTGGAATGT  R: CTCAACTGGTGTCGTGGAG |
